# Personalized Orthodontics: From the Sagittal Position of Lower Incisors to the Facial Profile Esthetics

**DOI:** 10.3390/jpm11080692

**Published:** 2021-07-22

**Authors:** Marcin Derwich, Liwia Minch, Maria Mitus-Kenig, Agata Zoltowska, Elzbieta Pawlowska

**Affiliations:** 1 ORTODENT, Specialist Orthodontic Private Practice in Grudziadz, 86-300 Grudziadz, Poland; 2 Limed, Specialist Orthodontic Private Practice in Wroclaw, 53-010 Wroclaw, Poland; liwiaminch@tlen.pl; 3 Department of Experimental Dentistry and Prophylaxis, Medical College, Jagiellonian University, 31-008 Krakow, Poland; maria.mitus@interia.pl; 4 Department of Endodontic Dentistry, Faculty of Medicine, Medical University of Gdansk, 80-210 Gdansk, Poland; azolt@gumed.edu.pl; 5 Department of Orthodontics, Medical University of Lodz, 90-419 Lodz, Poland; elzbieta.pawlowska@umed.lodz.pl

**Keywords:** facial profile esthetics, facial profile convexity, orthodontics, incisor inclination, personalized orthodontics

## Abstract

Background: One of the goals of orthodontic treatment is to obtain maximum facial harmony. The sagittal position of the lower incisors plays a significant role in the planning of orthodontic treatment. The aim of the study was to evaluate the relationship between the sagittal position of lower incisors and facial profile esthetics with reference to the skeletal vertical dimension. Methods: There were 200 patients included in the study. Patients were allocated into three groups, depending on the vertical growth pattern: normal-angle, low-angle, and high-angle cases. Tweed–Merrifield cephalometric analysis was used to assess the sagittal and vertical position of the mandible, as well as to assess the sagittal position of the lower incisors. Results: Z-angle and Frankfort mandibular incisor plane angle (FMIA) decreased significantly (*p* < 0.001) with the increase of the skeletal vertical dimension. Incisor mandibular plane angle (IMPA) was significantly higher (*p* < 0.001) in low-angle patients compared to the high-angle ones. Z-angle appeared to be the most accurate parameter (area under curve, AUC = 0.957) describing patients with a convex profile. The cutoff value of Z-angle 68.0° was characterized by the sensitivity of 94.1% and the specificity of 84.3%. Conclusions: The sagittal position of the lower incisors significantly affects the facial profile convexity. The Z-angle is the parameter which most accurately describes the patients with a convex profile.

## 1. Introduction

The sagittal position of the lower incisors plays a significant role in the planning of orthodontic treatment [1,2]. Several different aspects related to the position of the lower incisors have been discussed in the literature. These are: the amount of crowding and the dentoalveolar discrepancy [3,4], the long-term stability of orthodontic treatment [5,6,7,8], the relationship between the lower incisors’ position and the presence of gingival recessions [9,10,11], and finally, the facial profile esthetics [6,12,13,14]. Obtaining maximum facial harmony is one of the most important goals of orthodontic treatment [13].

Margolis [15] was the first one who analyzed the relationship between the long axis of the mandibular incisor and the base of the mandible, known as the incisor mandibular plane angle (IMPA). However, Tweed [16,17,18] noticed that both the occlusion and facial esthetics were interdependent. He constructed the diagnostic facial triangle [19], the valuable tool used for cephalometric analysis, with the three lines: the Frankfort horizontal plane, the mandibular plane, and the long axis of the mandibular incisor. There are three angles within the facial triangle between the abovementioned lines: the Frankfort mandibular plane angle (FMA), Frankfort mandibular incisor plane angle (FMIA), and the previously mentioned IMPA [19,20,21]. Tweed emphasized that in cases of class I malocclusion, class II malocclusion, and bimaxillary protrusion (all cases without severe skeletal discrepancies), to achieve the balanced facial esthetics, the recommended value of an IMPA angle was 90° ± 5° [16,20]. However, according to further observations by Tweed, the FMIA angle appeared to have the most important impact on facial esthetics. The rule of thumb was to achieve an FMIA of 65°–68° at the end of orthodontic treatment. These values were found to be associated with the best facial results [20,21].

Merrifield [13] introduced the term of the profile line, which connected the most prominent point on soft-tissue chin with the most prominent point on either the upper or lower lip, depending on which lip was more protruded. The profile line was extended to the Frankfort horizontal plane. The angle between the profile line and the Frankfort horizontal plane was named the Z-angle, and its recommended value was from 75° to 78° [20]. The aim of the profile line was to present the amount of lip protrusion [13]. Facial profile convexity may be easily assessed by the distance of the profile line from the tip of the nose. The more anteriorly from the tip of the nose the profile line localized is, the more convex the profile is. The well-balanced facial profile is characterized by the profile line crossing the nose [12].

The orthodontic achievements and clinical observations of Dr. Charles Tweed, as well as the results of further studies performed by the members of the Charles H. Tweed International Foundation for Orthodontic Research, including the studies by Dr. Levern Merrifield, were used to prepare the differential diagnostic analysis system [20,22]. Differential diagnosis includes skeletal, dental, and facial diagnosis. Moreover, it respects the anterior, posterior, lateral, and vertical limits of the dentition [20].

The aim of the study was to evaluate the relationship between the sagittal position of the lower incisors and facial profile esthetics with reference to the skeletal vertical dimension. The null hypothesis was that the sagittal position of the lower incisors does not affect the facial profile esthetics.

## 2. Materials and Methods

The Medical Board Ethical Committee (KB-18/21) approved the study. The study was conducted with the ethical principles of the World Medical Association Declaration of Helsinki. All patients received and signed informed consent.

There were 200 generally healthy patients included in the study, who had come for a specialist orthodontic consultation. The specialist orthodontic consultation was performed in the specialist orthodontic private practice in Grudziadz (Poland). None of the included patients had ever been treated orthodontically with either a removable or fixed orthodontic appliance.

Table 1 presents inclusion and exclusion criteria for the examined patients.

All of the patients who had been included in the study underwent orthodontic examination. Specialist orthodontic examination included: medical history, extraoral and intraoral examination, analysis of extraoral and intraoral photographs, analysis of plaster casts and analysis of radiographs, and both the lateral cephalograms (taken in the natural head position) and dental panoramic tomograms. The radiographs were taken on MyRay Hyperion X9 3D (Cefla, Imola, Italy).

Tweed–Merrifield cephalometric analysis was used to assess the sagittal and vertical position of the mandible, as well as to assess the sagittal position of the lower incisors [16,17,18,19,20,21,22]. We used Wits analysis to measure the sagittal jaw relationship. The FMA angle was used to assess the vertical position of the mandible with reference to the Frankfort horizontal plane. FMIA and IMPA angles were used to measure the sagittal position of the lower incisors.

**Figure 1:**
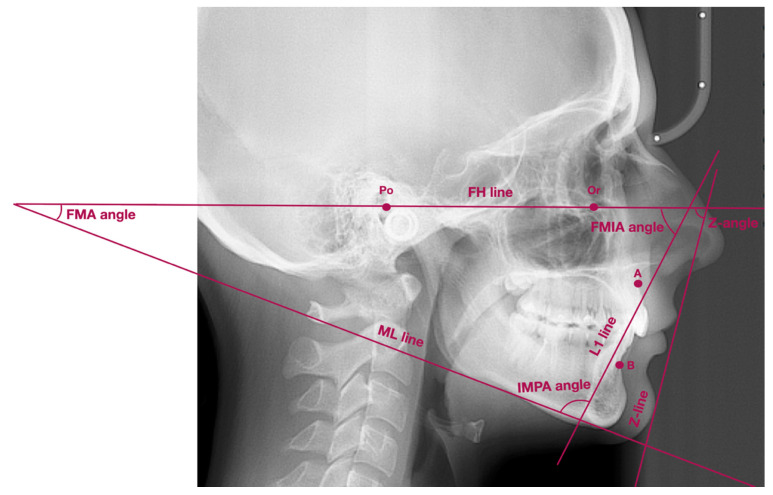
Lateral cephalogram with marked points, lines, and angles presented in Table 2. FMA—Frankfort mandibular plane angle, FMIA—Frankfort mandibular incisor plane angle, IMPA—incisor mandibular plane angle, ML line—mandibular line, FH line—Frankfort horizontal line, L1 line—long axis of lower incisor, Z-line—the profile line.

Figure 1 presents the lateral cephalogram with marked lines and angles, presented in Table 2.

Facial profile convexity was assessed by the distance of the Z-line from the tip of the nose. The facial profile was classified as: convex when the Z-line was moved forward from the tip of the nose, normal when the Z-line was crossing the nose, and borderline when the Z-line was crossing the tip of the nose. The Z-angle was used to measure the convexity of the facial profile.

All patients were allocated into groups, depending on the vertical growth pattern, estimated with the FMA angle. There were three groups included in the study: normal-angle cases (control group) with an FMA angle from 22° to 28° (76 patients), low-angle cases (group 1) with an FMA angle below 22° (51 patients), and high-angle cases (group 2) with an FMA angle above 28° (73 patients).

Statistical analysis was performed with Statistica 13.0 software (Dell Inc., Aliso Viejo, CA, USA). Quantitative variables were characterized with the usage of mean values, standard deviation, range, median, and upper and lower quartiles. To check if the differences between the examined groups were statistically significant, the analysis of variance (ANOVA) and chi-square test were used. The statistical significance level was set at *p* = 0.05.

## 3. Results

We estimated the sample size on the basis of an initial pilot study. We considered a power of 80% and a probability of the type I error 0.05. At least 50 patients in each group were necessary in order to detect a 20% difference in the Z-angle value between the groups.

### 3.1. Demographic Characteristics of the Examined Patients

There were 200 patients (148 females, 52 males; median age: 17 years; range: 15–25) included in the study. The control group consisted of 55 females and 21 males (median age: 17 years; range: 15–25). Group 1 (low-angle cases) consisted of 37 females and 14 males (median age: 17 years; range: 15–25). Finally, Group 2 (high-angle cases) consisted of 56 females and 17 males (median age: 17 years; range: 15–25).

Table 3 presents a comparison of age and sex among the examined groups.

High-angle patients were significantly younger compared to the low-angle and control groups. There were no statistically significant differences among the examined groups regarding the sex distribution.

### 3.2. Cephalometric Assessment of the Sagittal Jaw Relationship

The examined groups differed significantly regarding the sagittal jaw relationship. The majority of patients in the control group (46.1%) and in Group 2 (45.2%) were diagnosed with skeletal class I. However, the vast majority of low-angle patients (56.9%) presented with skeletal class II. The distribution of skeletal class II was significantly higher in Group 1 compared to the control group (56.9% vs. 34.2%; *p* = 0.013) and also compared to Group 2 (56.9% vs. 27.4%; *p* = 0.001). The distribution of skeletal class III was significantly higher in Group 2 compared to Group 1 (27.4% vs. 9.8%; *p* = 0.018).

The average values of the Wits analysis were significantly higher in Group 1 compared to the control group (*p* < 0.05), as well as compared to Group 2 (*p* < 0.01). There were no statistically significant differences regarding the average values of the Wits analysis between the control group and Group 2 (*p* > 0.05).

**Figure 2:**
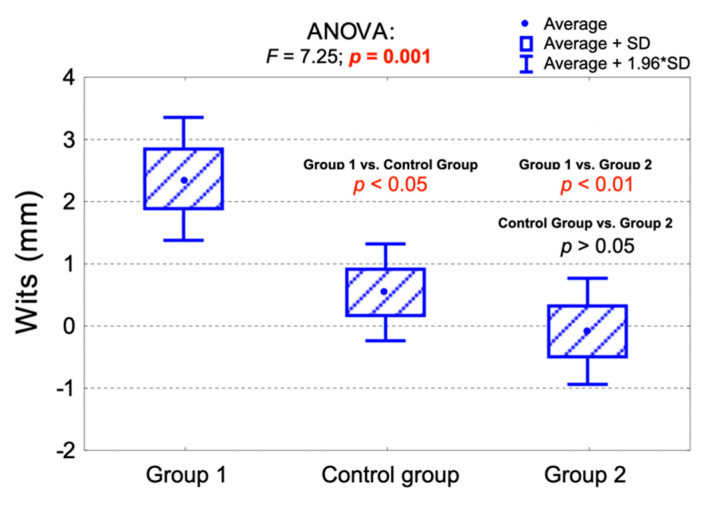
Comparison of the average Wits values among the examined groups and the results of the analysis of variance (ANOVA).

Table 4 presents the Wits analysis among the examined groups. Figure 2 presents the comparison of the average Wits values among the examined groups and the results of the analysis of variance (ANOVA).

### 3.3. Analysis of the Facial Profile Convexity

The examined groups differed significantly regarding the facial profile convexity. The distribution of the convex profile was significantly higher in Group 2 compared to the control group (28.8% vs. 11.8%; *p* = 0.011), as well as comparing to Group 1 (28.8% vs. 7.8%; *p* = 0.005). Of all convex profiles, 61.8% were diagnosed among high-angle cases.

The average value of the Z-angle was significantly higher in Group 1 compared to the control group (*p* < 0.01), as well as comparing to Group 2 (*p* < 0.001). Moreover, the average value of the Z-angle was significantly higher in the control group compared to Group 2 (*p* < 0.001). High-angle patients (group 2) presented the lowest values of the Z-angle.

**Figure 3:**
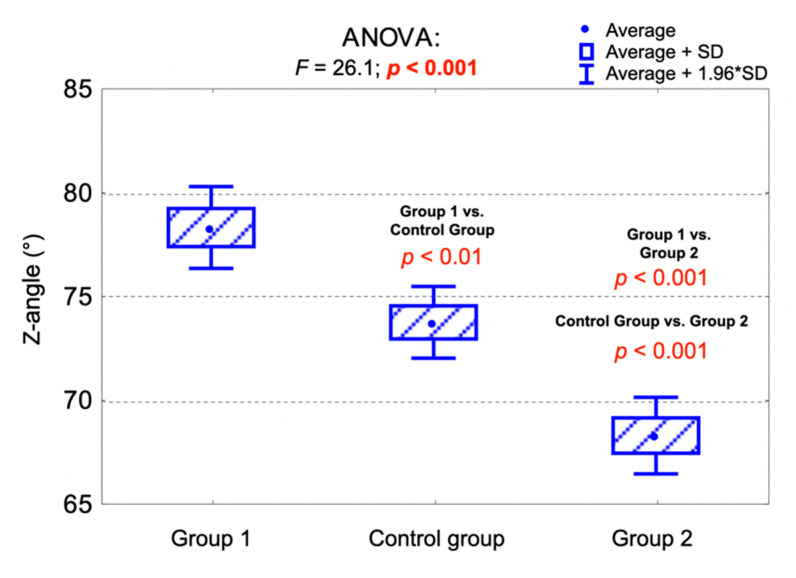
Comparison of the average Z-angle values among the examined groups and the results of the analysis of variance (ANOVA).

Table 5 presents the distribution of the Z-line position and the values of the Z-angle among the examined groups. Figure 3 presents the comparison of the average Z-angle values among the examined groups and the results of the analysis of variance (ANOVA).

Patients diagnosed with Z-line crossing the nose (normal profile) presented a significantly higher average value of the Z-angle compared to the patients diagnosed with a convex profile—Z-line in front of the nose (75.9° vs. 60.5°, *p* < 0.001), as well as compared to the patients diagnosed with a borderline profile—Z-line crossing the tip of the nose (75.9° vs. 66.5°, *p* < 0.01). The differences regarding the Z-angle average value between the patients with convex and borderline profiles were statistically insignificant.

Table 6 presents the average values of the Z-angle with reference to the position of the Z-line.

### 3.4. Assessment of the Cephalometric Angles: FMIA, IMPA

There were statistically significant differences between the examined groups regarding the average values of the IMPA and FMIA angles.

The average value of the IMPA angle was significantly lower in Group 2 compared to Group 1 (*p* < 0.01). The differences regarding the average value of the IMPA angle between the control group and Group 1, as well as between the control group and Group 2, were statistically insignificant (*p* > 0.05).

Group 2 presented the lowest average values of the FMIA angle. The average value of the FMIA angle was significantly lower in Group 2 compared to the control group (*p* < 0.001), as well as comparing to Group 1 (*p* < 0.001). Moreover, the average value of the FMIA angle was significantly lower in the control group compared to Group 1 (*p* < 0.01).

**Figure 4:**
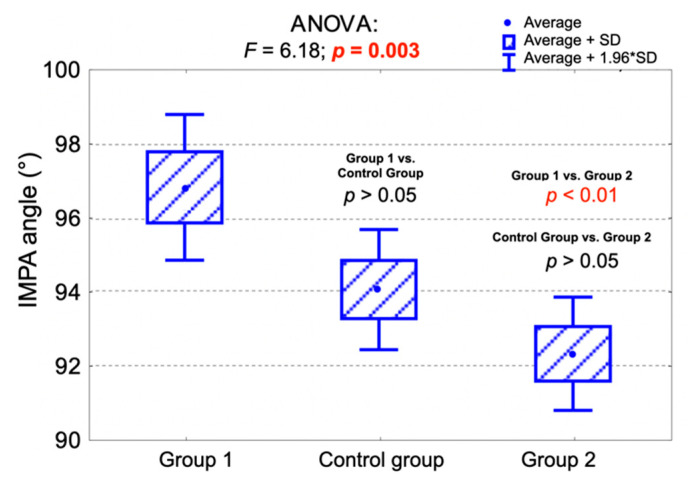
Comparison of the average IMPA angle values among the examined groups and the results of the analysis of variance (ANOVA). IMPA—incisor mandibular plane angle.

**Figure 5:**
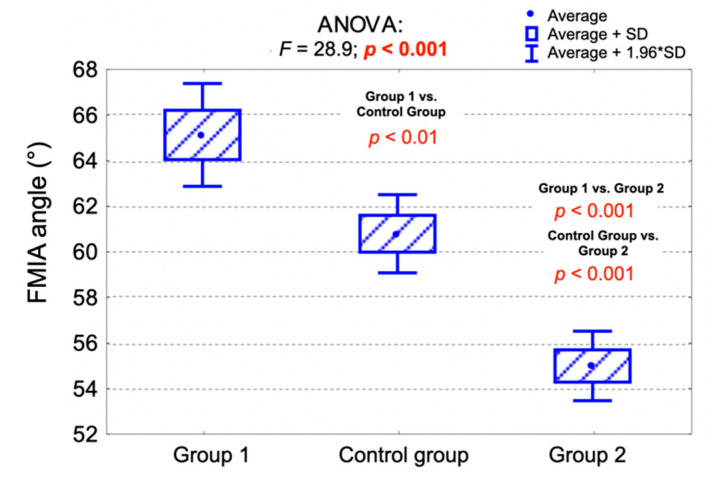
Comparison of the average FMIA angle values among the examined groups and the results of the analysis of variance (ANOVA). FMIA—Frankfort mandibular incisor plane angle.

Table 7 presents the average values of the FMIA and IMPA angles among the examined groups. Figure 4 and Figure 5 present the comparison of the average IMPA angle values (Figure 4) and FMIA angle values (Figure 5) among the examined groups and the results of the analysis of variance (ANOVA).

Patients diagnosed with Z-line crossing the nose (normal profile) presented a significantly higher average value of the FMIA angle compared to the patients diagnosed with a convex profile—Z-line in front of the nose (62.3° vs. 49.6°, *p* < 0.001), as well as compared to the patients diagnosed with a borderline profile—Z-line crossing the tip of the nose (62.3° vs. 53.1°, *p* < 0.01). Moreover, the average value of the IMPA angle was significantly lower in patients with a normal profile compared to patients with a convex profile (92.4° vs. 100.6°, *p* < 0.001).

Table 8 presents the average values of the FMIA and IMPA angles with reference to the position of the Z-line.

**Figure 6:**
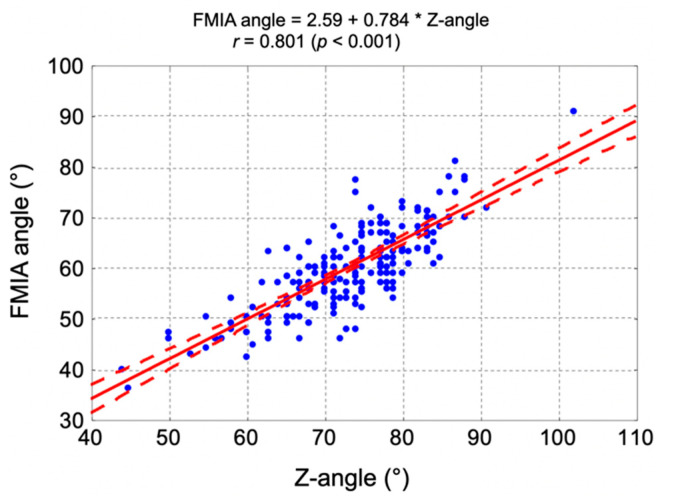
The correlation diagram between the Z-angle and FMIA angle, correlation coefficient, and regression straight-line equation. FMIA—Frankfort mandibular incisor plane angle.

There was a statistically significant positive correlation between the Z-angle and FMIA angle (*r* = 0.801, *p* < 0.001). Figure 6 presents the correlation diagram between the Z-angle and FMIA angle, correlation coefficient, and regression straight-line equation.

**Figure 7:**
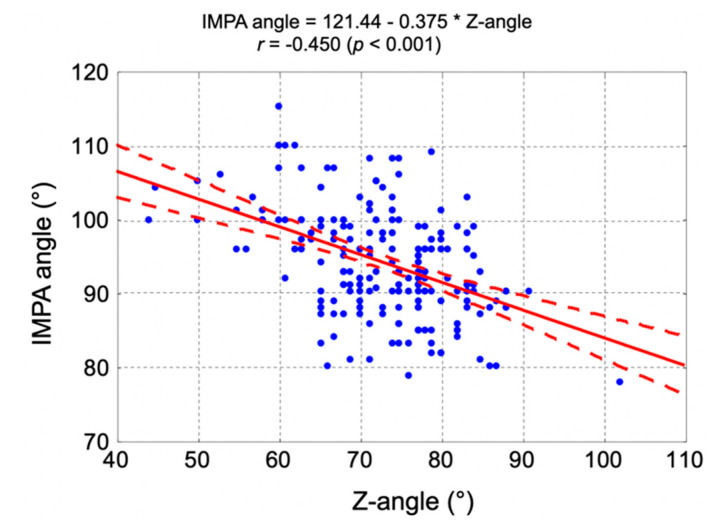
The correlation diagram between the Z-angle and IMPA angle, correlation coefficient, and regression straight-line equation. IMPA—incisor mandibular plane angle.

There was also a statistically significant negative correlation between the Z-angle and IMPA angle (*r* = −0.450, *p* < 0.001). Figure 7 presents the correlation diagram between the Z-angle and IMPA angle, correlation coefficient, and regression straight-line equation.

### 3.5. Cephalometric Parameters Describing Patients with Convex Profile

Receiver operating characteristic (ROC) curves were used to find the most accurate cephalometric parameters describing patients with a convex profile. Table 9 presents the results of the analysis of the ROC curves.

**Figure 8:**
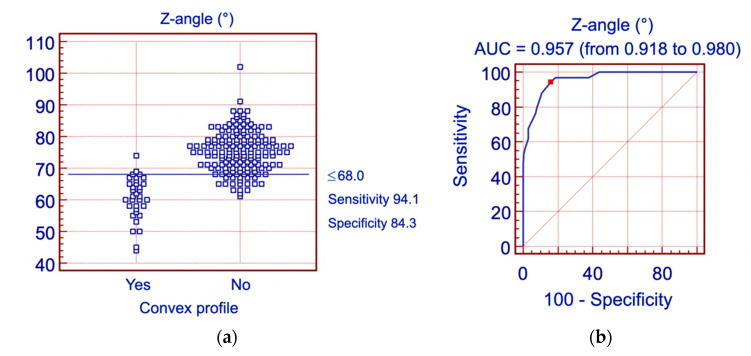
Analysis of the Z-angle with reference to the profile convexity: (**a**) Histogram; (**b**) ROC curve for the Z-angle. AUC—area under curve.

**Figure 9:**
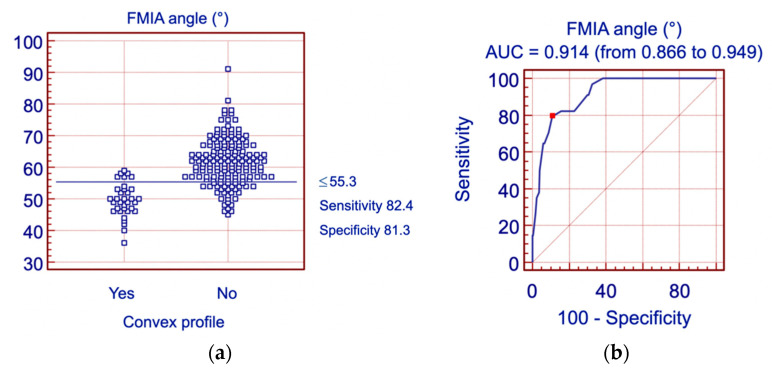
Analysis of the FMIA angle with reference to the profile convexity: (**a**) Histogram; (**b**) ROC curve for the FMIA angle. FMIA – Frankfort mandibular incisor plane angle, AUC—area under curve.

The Z-angle appeared to be the most accurate parameter (AUC = 0.957) describing patients with a convex profile. The cutoff value Z-angle ≤ 68.0° was characterized by the sensitivity of 94.1% and the specificity of 84.3%. The second most accurate parameter describing patients with a convex profile was the FMIA angle (AUC = 0.914). The cutoff value FMIA ≤ 55.3° was characterized by the sensitivity of 82.4% and the specificity of 81.3%. Figure 8a,b and Figure 9a,b present the histogram and ROC curve for the Z-angle and FMIA angle.

## 4. Discussion

This study evaluates the relationship between the sagittal position of the lower incisors and facial profile esthetics with reference to the skeletal vertical dimension. Moreover, according to our knowledge, this is the only study which analyzes different cephalometric parameters to find these parameters which most accurately describe patients with a convex profile.

We analyzed a group of 200 patients who had been allocated into one of three subgroups, depending on the skeletal vertical dimension. We have found statistically significant differences between the groups with reference to the sagittal jaw relationship (based on Wits analysis). Patients diagnosed with FMA < 22° most often presented a skeletal class II relationship. There were no statistically significant differences between normal-angle and high-angle patients regarding the sagittal jaw relationship. Interestingly, Linjawi [24] noticed that only patients with a decreased skeletal vertical dimension presented changes in the inclination and position of the lower incisors related to age and gender.

The majority of patients included in our study, who were diagnosed with a convex profile, presented an increased vertical dimension. We have observed that Z and FMIA angles’ values decreased significantly with the increase of the skeletal vertical dimension. Both the Z and FMIA angles were measured with reference to the Frankfort horizontal line (FH line). Thus, with the increase of the skeletal vertical dimension and posterior rotation of the mandible, we observed the decrease in the values of the Z and FMIA angles. This was also related with the increased facial profile convexity.

Conversely, the IMPA angle was significantly higher in low-angle patients compared to the high-angle ones. There were no other significant differences between the examined groups. The IMPA angle describes the sagittal position of the lower incisor with reference to the mandibular line. The above-described difference of the IMPA values can be explained with the process of the dentoalveolar compensation of the skeletal types of malocclusions. Alhammadi [25] described that there is an association between the sagittal jaw discrepancy and the sagittal position of the incisors; namely, he observed a proclination of the lower incisors with an increased sagittal jaw relationship (or increased overjet). He also described the significant negative correlation between the skeletal vertical dimension and inclinations of the maxillary and mandibular incisors. This means that with increased vertical dimension, both the upper and lower incisors become more retroclined.

Furthermore, we have found that the facial convexity was most accurately described with the Z-angle measurement. The value of the Z-angle ≤ 68.0° characterizes the patients with a convex profile with a sensitivity of 94.1% and specificity of 84.3%. The second parameter most accurately describing patients with a convex profile was the FMIA angle ≤ 55.3° with a sensitivity of 82.4% and specificity of 81.3%.

Facial analysis is the onset of orthodontic diagnosis [26]. Facial profile esthetics depend on several different factors, including, among others: facial anatomy, the skeletal vertical and horizontal position of the maxilla and mandible, dentoalveolar sagittal position, thickness of lips, activity of facial muscles, and ethnicity [27]. The vast majority of the available manuscripts discuss the relationship between the facial profile convexity and the proclination of incisors in terms of bimaxillary protrusion treatment. It is generally known that the extraction of four premolars, especially the first ones, creates spaces to straighten and retract the anterior teeth. This approach allows correction of dentoalveolar protrusion, and therefore leads to improvement of facial balance [27,28,29]. Contini et al. [30] indicated that the facial balance is not only affected by orthodontic treatment, but also by facial growth. Krooks et al. [31] performed esthetic evaluation of facial attractiveness. They found that facial sagittal dimensions affected more facial esthetics than the vertical dimensions.

The sagittal position of the lower incisors should always be assessed with reference to the skeletal vertical dimension. To obtain the harmony of the facial profile, hyperdivergent patients require a more upright position of the lower incisors than the hypodivergent patients do. Dr. Tweed’s formula recommends, for patients with FMA from 21° to 29°, to obtain the FMIA angle of 68°; for patients with FMA 30°, the FMIA should be 65°; and finally, for patients with FMA 20° or less, the IMPA angle should not exceed 92° [2].

A few factors affecting the position of the mandibular incisors have been listed, namely: natural oral function, facial harmony, and support by the periodontal tissues [32]. The final position of the lower incisors is the result of the abovementioned factors [32]. However, not only do the periodontal tissues affect the position of the lower incisors, but the position of the lower incisors also affects the condition of the periodontal tissues. Proclination of the lower incisors may lead to reduction of gingival thickness, shortening of the free gingival margin, and may also lead to increased clinical crown height [33]. Hasegawa et al. [32] found that there was no relationship between the morphology of the upper incisors (labiolingual crown thickness of maxillary incisors) and the position of the mandibular incisors. Gütermann et al. [34] noticed that inclination of lower incisors is associated with sex, age, and the skeletal vertical pattern. Furthermore, according to their studies, there is only one symphysial distance—symphysial depth—which is related to inclination of the lower incisors. Finally, the position of the tongue and length of the lingual frenum may affect the shape of the dental arch, and consequently change the position of the incisors [35,36]. It has also been proven that the orofacial balance may affect the posture of the entire body [37].

We have found only one clinical study which assessed the relationship between repositioning of the lower incisors during orthodontic treatment and enhancement of the profile in patients with different vertical growth patterns. The study was performed by Contini et al. [30]. The authors compared the clinical outcomes of the orthodontic treatment in two groups of patients with different vertical growth patterns. Contini et al. found that patients with unfavorable growth patterns presented bigger changes in FMIA angles than patients with favorable growth patterns. Changes in the values of the Z-angle were similar in both groups. Contini et al. observed improvements in facial profile esthetics in both groups of patients. The authors indicated that the increase of the anterior facial height is related to the increase in facial profile convexity, and that the retraction of lower incisors (increase of FMIA angle) leads to lip retraction (increase of Z-angle), and therefore improves facial balance. Contini et al. confirmed that the position of the lower incisors affects the facial profile esthetics, and therefore, the facial profile esthetics may be improved with orthodontic treatment. Our observations stay in accordance with the research by Contini et al. [30].

There are a few limitations to our study. Firstly, this study was based only on people aged 15–25 years old. Therefore, the results from this study should not be generalized to the population of elderly patients. Facial profile esthetics in elderly patients is strongly affected by the process of aging. That is why further studies ought to be performed to assess the relationship between the sagittal position of the lower incisors and facial profile esthetics in different age groups. Secondly, we did not analyze patients’ body mass index (BMI). It may be speculated that obesity may affect the value of the Z-angle. Finally, we did not evaluate patients’ satisfaction related to their facial profile. It should be remembered that there may exist differences in the perception of facial esthetics between patients and orthodontic specialists.

## 5. Conclusions

The sagittal position of the lower incisors significantly affects facial profile convexity. The Z-angle and FMIA angle are the parameters which most accurately describe the patients with convex profiles. We found a strong positive correlation between the Z-angle and the FMIA angle. The soft tissue facial profile indicates the sagittal position of the anterior limit of the dentition.

## Figures and Tables

**Table 1:** Inclusion and exclusion criteria for the examined patients.

Criteria	List of Specific Criteria
Inclusion criteria	- Generally healthy patients (no systemic diseases) - Age between 15 and 25 years old - Willingness to participate in the study - No previous orthodontic treatment
Exclusion criteria	- Age below 15 and above 25 years old - History of traumas in the area of head and neck - History of surgeries in the area of head and neck - Craniofacial deformities - Cleft lip and cleft palate- Genetic syndromes - Temporomandibular joint disorders - Rheumatological diseases, oncological diseases - History of radiotherapy (especially in the area of the head and neck) - People treated orthodontically at least once in the past - Pregnancy - Patients who did not agree to take part in the study

**Table 2:** List of points, lines, and angles used to analyze the mandibular position and morphology, as well as the position of the lower incisors on the basis of the literature [16,17,18,19,20,21,22,23].

Points/Lines/Angles	Description of Points/Lines/Angles
Point Po	Porion—the most superior part of external acoustic opening
Point Or	Orbitale—the most inferior point localized in the lower margin of the orbit
Point A	Subspinale—the deepest point localized in the anterior outline of the maxilla, below the anterior nasal spine
Point B	Supramentale—the deepest point localized in the anterior outline of the mandible, above the pogonion
Wits	AO-BO—distance between the perpendicular projection of points A and B onto the functional occlusal plane
ML line	Mandibular line—line between gnathion and the lowest point localized in the masseteric tuberosity (also known as GoGn line)
FH line	Frankfort horizontal line—line between points: porion and orbitale
L1 line	Long axis of lower incisor—line which connects the incisal edge with the radiological apex of lower incisor
Z-line	Z-line (the profile line)—line which connects the most prominent point on soft-tissue chin with the most prominent point on either upper or lower lip, depending on which lip was more protruded
FMA angle	Angle between FH line and ML line
FMIA angle	Angle between FH line and long axis of lower incisor
IMPA angle	Angle between long axis of lower incisor and ML line
Z-angle	Angle between Z-line and FH line

FMA—Frankfort mandibular plane angle, FMIA—Frankfort mandibular incisor plane angle, IMPA—incisor mandibular plane angle, ML line—mandibular line, FH line—Frankfort horizontal line, L1 line—long axis of lower incisor, Z-line—the profile line.

**Table 3:** Comparison of age and sex among the examined groups.

Comparable Characteristic	Control group (*n* = 76) FMA 22°–28°	Group 1 (*n* = 51) FMA < 22°	Group 2 (*n* = 73) FMA > 28°	Together (*n* = 200)	*p*-Value
AGE					0.006 ^1^
av. (SD)	18.6 (3.5)	18.5 (3.2)	17.1 (2.8)	18.0 (3.2)	
range	15.0–25.0	15.0–25.0	15.0–25.0	15.0–25.0	
median (Q_1_;Q_3_)	17 (15;20)	17 (15;21)	16 (15;18)	17 (15;20)	
SEX					0.802 ^2^
Female (%)	55 (72.4%)	37 (72.5%)	56 (76.7%)	148 (74.0%)	
Male (%)	21 (27.6%)	14 (27.5%)	17 (23.3%)	52 (26.0%)	

^1^ Kruskal-Wallis test; ^2^ Chi-square test; av.—average; SD—standard deviation; Q_1_—lower quartile; Q_3_—upper quartile, FMA – Frankfort mandibular plane angle.

**Table 4:** Wits analysis among the examined groups.

Measurement	Control Group (*n* = 76) FMA 22°–28°	Group 1 (*n* = 51) FMA < 22°	Group 2 (*n* = 73) FMA > 28°	Together (*n* = 200)	*p*-Value
Wits analysis					0.010 ^1^
Skeletal class I (Wits = 0 ± 2 mm)	35 (46.1%)	17 (33.3%)	33 (45.2%)	85 (42.5%)	
Skeletal class II (Wits > 2 mm)	26 (34.2%)	29 (56.9%)	20 (27.4%)	75 (37.5%)	
Skeletal class III (Wits < −2 mm)	15 (19.7%)	5 (9.8%)	20 (27.4%)	40 (20.0%)	
Wits (mm)					0.001 ^2^
av. (SD)	0.5 (3.5)	2.3 (3.6)	−0.1 (3.7)	0.8 (3.7)	
range	−7.0–8.0	−9.0–9.0	−9.0–9.0	−9.0–9.0	
median (Q_1_;Q_3_)	0 (−2;3)	3 (0;5)	0 (−2;2)	1 (−1;4)	

^1^ Chi-square test; ^2^ ANOVA; av.—average; SD—standard deviation; Q_1_—lower quartile; Q_3_—upper quartile, FMA – Frankfort mandibular plane angle.

**Table 5:** Distribution of the Z-line position and the values of the Z-angle among the examined groups.

Measurement	Control Group (*n* = 76) FMA 22°–28°	Group 1 (*n* = 51) FMA < 22°	Group 2 (*n* = 73) FMA > 28°	Together (*n* = 200)	*p*-Value
Z-line position					0.003 ^1^
In front of the nose (convex profile)	9 (11.8%)	4 (7.8%)	21 (28.8%)	34 (17.0%)	
Crossing the nose (normal profile)	61 (80.3%)	47 (92.2%)	48 (65.8%)	156 (78.0%)	
Crossing the tip of the nose (borderline profile)	6 (7.9%)	0 (0.0%)	4 (5.5%)	10 (5.0%)	
Z-angle (°)					<0.001 ^2^
av. (SD)	73.7 (7.7)	78.2 (7.2)	68.2 (8.0)	72.8 (8.6)	
range	50.0–87.0	60.0–102.0	44.0–82.0	44.0–102.0	
median (Q_1_;Q_3_)	75 (68;79)	78 (74;83)	69 (65;75)	74 (68;79)	

^1^ Chi-square test; ^2^ ANOVA; av.—average; SD—standard deviation; Q_1_—lower quartile; Q_3_—upper quartile, FMA—Frankfort mandibular plane angle.

**Table 6:** The average values of the Z-angle with reference to the position of the Z-line.

Measurement	Convex Profile (*n* = 34) Z-Line in front of the Nose	Borderline Profile (*n* = 10) Z-Line Crossing the Tip of the Nose	Normal Profile (*n* = 156) Z-LineCrossing the Nose	*p*-Value
Z-angle (°)				<0.001 ^1^
av. (SD)	60.5 (6.8)	66.5 (3.8)	75.9 (6.2)	
range	44.0–74.0	61.0–70.0	63.0–102.0	
median (Q_1_;Q_3_)	61 (57;65)	68 (63;70)	76 (71;80)	

^1^ ANOVA; av.—average; SD—standard deviation; Q_1_—lower quartile; Q_3_—upper quartile.

**Table 7:** The average values of the FMIA and IMPA angles among the examined groups.

Measurement	Control Group (*n* = 76) FMA 22°–28°	Group 1 (*n* = 51) FMA < 22°	Group 2 (*n* = 73) FMA > 28°	Together (*n* = 200)	*p*-Value
IMPA (°)					0.003 ^1^
av. (SD)	94.0 (7.2)	96.8 (7.2)	92.3 (6.7)	94.1 (7.2)	
range	80.0–110.0	78.0–115.0	79.0–106.0	78.0–115.0	
median (Q_1_;Q_3_)	94 (89;98)	96 (91;101)	92 (87;97)	93 (90;99)	
FMIA (°)					<0.001 ^1^
av. (SD)	60.7 (7.6)	65.1 (8.2)	54.9 (6.6)	59.7 (8.4)	
range	42.0–78.0	50.0–91.0	36.0–72.0	36.0–91.0	
median (Q_1_;Q_3_)	61 (56;67)	65 (60;70)	56 (50;59)	60 (54;65)	

^1^ ANOVA; av.—average; SD—standard deviation; Q_1_—lower quartile; Q_3_—upper quartile, FMA—Frankfort mandibular plane angle, FMIA—Frankfort mandibular incisor plane angle, IMPA—incisor mandibular plane angle.

**Table 8:** The average values of the FMIA and IMPA angles with reference to the position of the Z-line.

Measurement	Convex Profile (*n* = 34) Z-Line in front of the Nose	Borderline Profile (*n* = 10) Z-Line Crossing the Tip of the Nose	Normal Profile (*n* = 156) Z-LineCrossing the Nose	*p*-Value
IMPA (°)				<0.001 ^1^
av. (SD)	100.6 (5.9)	98.1 (6.9)	92.4 (6.6)	
range	87.0–115.0	90.0–110.0	78.0–109.0	
median (Q_1_;Q_3_)	100 (97;104)	97 (92;103)	92 (88;97)	
FMIA (°)				<0.001 ^1^
av. (SD)	49.6 (5.4)	53.1 (6.0)	62.3 (7.1)	
range	36.0–59.0	45.0–62.0	49.0–91.0	
median (Q_1_;Q_3_)	50 (46;53)	54 (47;57)	62 (57;67)	

^1^ ANOVA; av.—average; SD—standard deviation; Q_1_—lower quartile; Q_3_—upper quartile, FMIA—Frankfort mandibular incisor plane angle, IMPA—incisor mandibular plane angle.

**Table 9:** The results of the analysis of the ROC curves.

Parameter	Cutoff Value	Sensitivity	Specificity	Area under the Curve (AUC)	95% CI for AUC
FMA angle	>29.0°	61.8%	72.3%	0.700	(0.632;0.763)
FMIA angle	≤55.3°	82.4%	81.3%	0.914	(0.866;0.949)
IMPA angle	>99.0°	67.6%	85.5%	0.815	(0.755;0.867)
Z angle	≤68.0°	94.1%	84.3%	0.957	(0.918;0.980)
Wits	>2.1 mm	58.8%	68.7%	0.657	(0.587;0.722)

FMIA—Frankfort mandibular incisor plane angle, IMPA—incisor mandibular plane angle, CI—confidence interval.

## Data Availability

The data underlying this article are available in the article.
